# Supplementary material for: SARS-CoV-2 viral entry and replication is impaired in Cystic Fibrosis airways due to ACE2 downregulation
Source: Nat Commun. 2023 Jan 10;14:132. doi: 10.1038/s41467-023-35862-0 (PMC9830623; doi:10.1038/s41467-023-35862-0)
Supplement: Supplementary file 1 — Supplementary Information [file 41467_2023_35862_MOESM1_ESM.pdf]

SARS-CoV-2 viral entry and replication is impaired in Cystic Fibrosis airways due to ACE2 downregulation

Valentino Bezzerri, Valentina Gentili, Martina Api, Alessia Finotti, Chiara Papi, Anna Tamanini, Christian Boni, Elena Baldisseri, Debora Olioso, Martina Duca, Erika Tedesco, Sara Leo, Monica Borgatti, Sonia Volpi, Paolo Pinton, Giulio Cabrini, Roberto Gambari, Francesco Blasi, Giuseppe Lippi, Alessandro Rimessi, Roberta Rizzo, and Marco Cipolli

Supplementary Table 1

Cell type	Sample ID	Sex	Age	Genetics (CFTR)
**CF**				
hNEC	CF-MD0408	N/A	24	Homozygote F508del
hNEC	CF-MD0803	N/A	25	Homozygote F508del
hBEC	CF-MD0502	M	25	Homozygote F508del
hBEC	CF-MD0673	F	23	Homozygote F508del
hBEC	CF-MD0567	F	39	Homozygote F508del
hBEC	CF- MD0679	F	30	Homozygote F508del
hBEC	CF- MD0519	F	33	Homozygote F508del
hBEC	CF- MD0607	F	21	Homozygote F508del
hBEC	CF- MD0208	F	21	Compound Heterozygote 2184insA/W1282X
**Control**				
hNEC	MP0009	N/A	pool*	normal
hBEC	MP0768	F	59	normal
hBEC	MD0742	M	61	normal
hBEC	MD0642	F	21	normal
hBEC	MD0670	M	15	normal
hBEC	MD0835	M	35	normal
hBEC	MD0812	M	42	normal
hBEC	MD0805	M	17	normal
hBEC	MD0834	M	71	normal

Supplementary Table 1. Genetics and clinical data of Mucilair® tissues (Epithelix) analyzed in this study. N/A, not available; F, female; * pool of hNEC isolated from 14 heathy donors.

Supplementary Fig.1

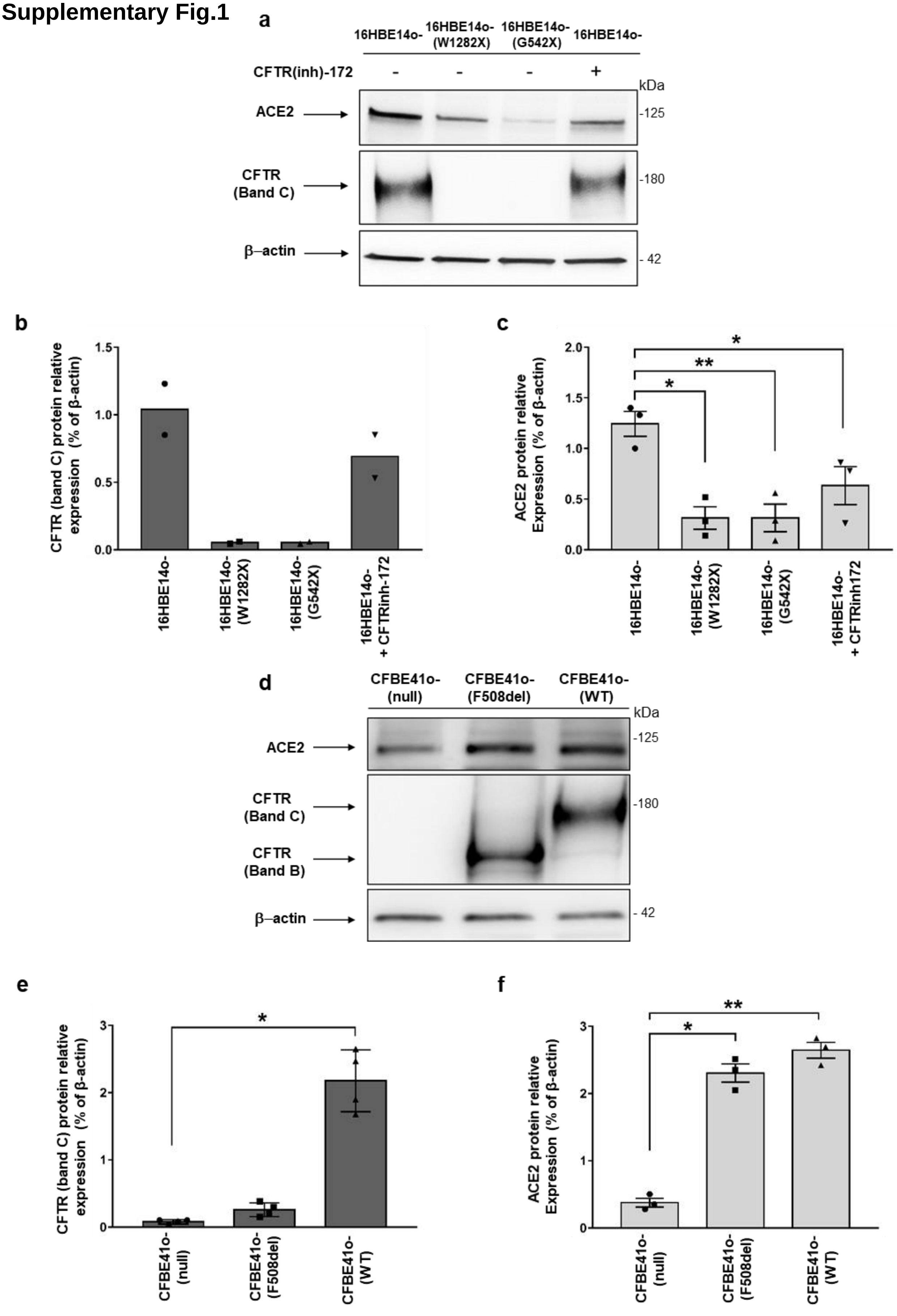

Supplementary Fig. 1.

CFTR expression regulates ACE2 protein levels in unpolarized bronchial epithelial cells. **a,** Representative western blot analysis of ACE2 and CFTR performed in protein extracts from unpolarized parental 16HBE14o- cells and 16HBE14o- cells carrying biallelic W1282X-*CFTR* or G542X-*CFTR* mutations in the presence (+) or absence (-) of 5 μM CFTR(inh)-172, for 24 h. The samples used to detect CFTR derived from the same experiments depicted in panel **a** but gels were processed in parallel. **b**, Densitometry analysis of CFTR (% of β-actin expression). Data are shown as mean ± SEM of two independent experiments (n=2). **c**, Densitometry analysis of ACE2 (% of β-actin expression). Data are shown as mean ± SEM of three independent experiments (n=3). **d**, Western blot analysis of ACE2 and CFTR performed in protein extracts from unpolarized CFBE41o- cells (null) and in cells expressing F508del-CFTR (F508del) or wild-type CFTR (WT). The samples used to detect CFTR derived from the same experiments depicted in panel **d** but gels were processed in parallel. **e**, Densitometry analysis of CFTR (% of β-actin expression). Data are shown as mean ± SEM of three independent experiments (n=3). **f**, Densitometry analysis of ACE2 (% of β-actin expression). Data are shown as mean ± SEM of four independent experiments (n=4). Normal distribution was tested by the Shapiro–Wilk test before running the two-tailed Student's t test for paired data, which has been reported within the scatter plot with bars (*$p<0.05$; **$p<0.01$).

Supplementary Fig.2

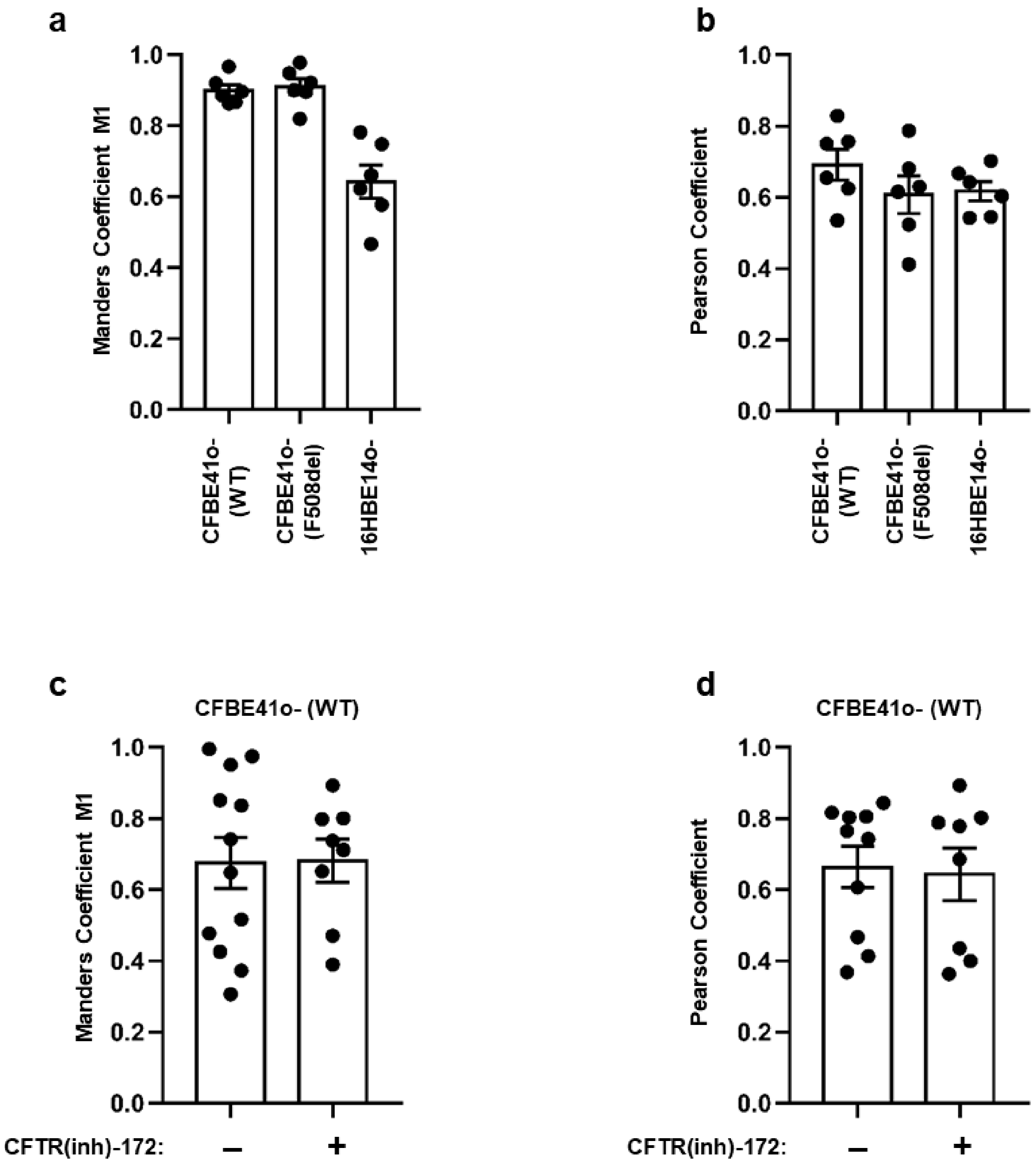

Supplementary Fig. 2.

Localization of CFTR on the plasma membrane, but not its function, is essential for the subcellular localization of ACE2 on the cell surface. **a**,**b** Quantification of the degree of colocalization between CFTR and the ACE2 receptor. Manders M1 (a) and Pearson's (b) coefficients represent the correlation between the intracellular localization of CFTR and ACE2 in the different cell lines (n=6). **c,d** Quantification of colocalization between CFTR and

ACE2 in CFBE41o-expressing wild-type *CFTR* (WT) in the presence (+) or absence (-) of 5 μM CFTR(inh)-172 for 24h. The scatter plot with bars shows the Manders M1 (c, n=12) and Pearson's (d, n=10) coefficients under different experimental conditions. Data represented in panels **a-d** are shown as mean ± SEM.

Supplementary Fig.3

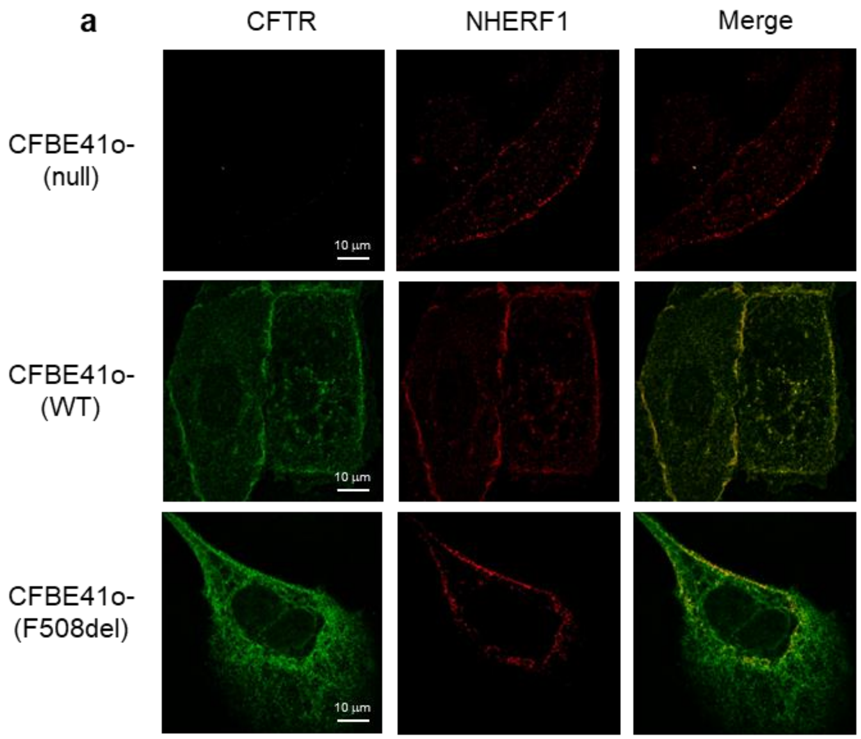

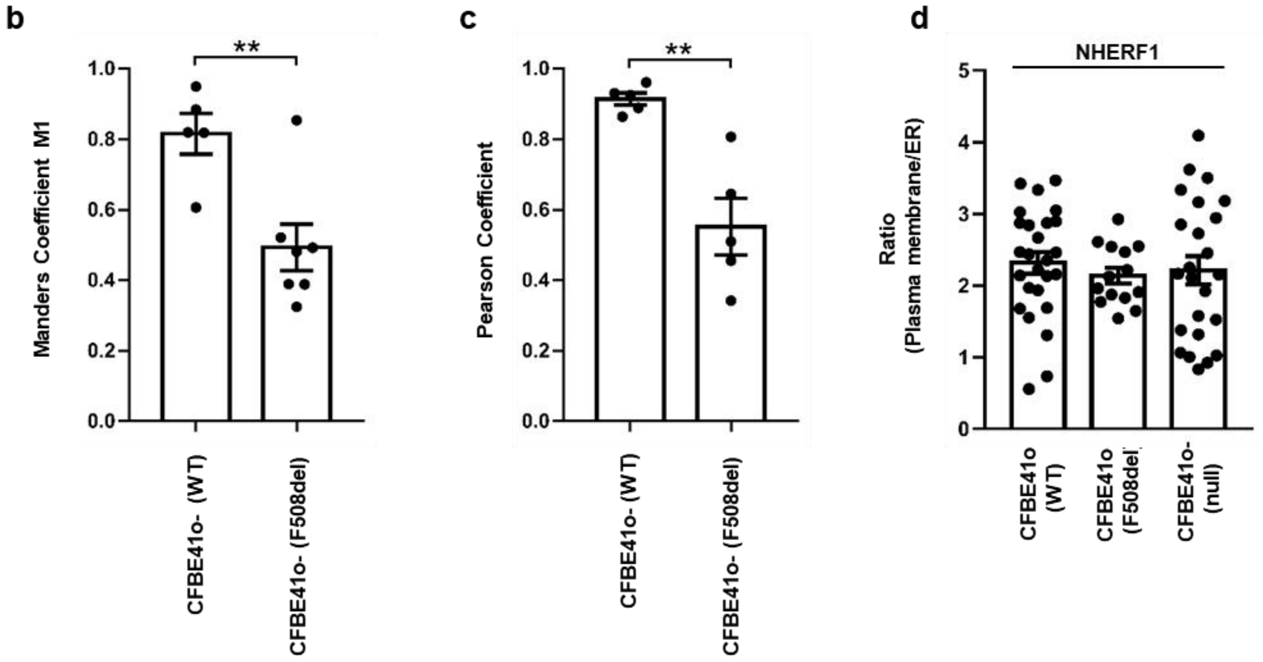

Supplementary Fig. 3.

CFTR subcellular localization does not affect localization of the NHERF1 scaffolding protein on the plasma membrane. a, Representative images of immunofluorescence detection of CFTR (green) and NHERF1 (red) under basal conditions in CFBE41o- (null), CFBE41o- over-expressing wild-type CFTR (WT), CFBE41o- over-expressing F508del-*CFTR* (scale bar: 10 μm). **b**-**d**, Quantification of colocalization between CFTR and NHERF1 and subcellular distribution of NHERF1 in different cell lines. Manders M1 (**b**, n=7) and Pearson's (**c**, n=5) coefficients represent the correlation between the intracellular localization of CFTR and NHERF1, whereas (**d**, n=26) represents the ratio between plasma membrane fluorescence and endoplasmic reticulum (ER) fluorescence of NHERF1. Data are mean ± SEM of 7 independent experiments. Normal distribution was tested by the Shapiro–Wilk test before running the two-tailed Student's t test for paired data, which has been reported within the scatter plot with bars (**p<0.01).
