# Supplementary material for: SARS-CoV-2 viral entry and replication is impaired in Cystic Fibrosis airways due to ACE2 downregulation
Source: Nat Commun. 2023 Jan 10;14:132. doi: 10.1038/s41467-023-35862-0 (PMC9830623; doi:10.1038/s41467-023-35862-0)
Supplement: Supplementary file 3 — Reporting Summary [file 41467_2023_35862_MOESM3_ESM.pdf]

nature portfolio

Corresponding author(s): Marco Cipolli

Last updated by author(s): Dec 28, 2022

Reporting Summary

Nature Portfolio wishes to improve the reproducibility of the work that we publish. This form provides structure for consistency and transparency in reporting. For further information on Nature Portfolio policies, see our Editorial Policies and the Editorial Policy Checklist.

Statistics

For all statistical analyses, confirm that the following items are present in the figure legend, table legend, main text, or Methods section.

n/a	Confirmed	
☐	☒	The exact sample size (*n*) for each experimental group/condition, given as a discrete number and unit of measurement
☐	☒	A statement on whether measurements were taken from distinct samples or whether the same sample was measured repeatedly
☐	☒	The statistical test(s) used AND whether they are one- or two-sided<br>*Only common tests should be described solely by name; describe more complex techniques in the Methods section.*
☒	☐	A description of all covariates tested
☐	☒	A description of any assumptions or corrections, such as tests of normality and adjustment for multiple comparisons
☐	☒	A full description of the statistical parameters including central tendency (e.g. means) or other basic estimates (e.g. regression coefficient) AND variation (e.g. standard deviation) or associated estimates of uncertainty (e.g. confidence intervals)
☐	☒	For null hypothesis testing, the test statistic (e.g. *F*, *t*, *r*) with confidence intervals, effect sizes, degrees of freedom and *P* value noted<br>*Give P values as exact values whenever suitable.*
☒	☐	For Bayesian analysis, information on the choice of priors and Markov chain Monte Carlo settings
☒	☐	For hierarchical and complex designs, identification of the appropriate level for tests and full reporting of outcomes
☒	☐	Estimates of effect sizes (e.g. Cohen's *d*, Pearson's *r*), indicating how they were calculated

Our web collection on statistics for biologists contains articles on many of the points above.

Software and code

Policy information about availability of computer code

Data collection	Western blot data were collected by ImageLab Touch (Bio-Rad, Version 3.0.1.14); immunofluorescence data were collected by Zeiss LSM510 confocal microscopy and using the Zeiss Zen 2009 (Version 6.0.0.303) software, and by Olympus FV3000 microscopy using Fluoview FV (Version 31s-sw); ELISA data were collected by Sunrise microplate reader (Tecan Trading AG, Männedorf, Switzerland); qPCR data were collected by GeneAmp PCR System 9700 thermal cycler (Thermo Fisher Scientific, Waltham, MA); dPCR data were collected by Automated Droplet Generator (AutoDG) (Bio-Rad, Hercules, CA) and QX200 Droplet Reader (Bio-Rad, Hercules, CA)
Data analysis	ImageJ (NIH, Bethesda, MD)(Version 1.8.0_172) for western blot and immunofluorescence, GraphPad Prism (GraphPad Software, San Diego CA)(Version 7) for statistical analysis, Qiaquant96 (QIAGEN, Hilden, Germany)(Version 1.0.3) for qPCR, QuantaSoft (QIAGEN, Hilden, Germany) (Version 1.7.4) for dPCR

For manuscripts utilizing custom algorithms or software that are central to the research but not yet described in published literature, software must be made available to editors and reviewers. We strongly encourage code deposition in a community repository (e.g. GitHub). See the Nature Portfolio guidelines for submitting code & software for further information.

Data

Policy information about availability of data

All manuscripts must include a data availability statement. This statement should provide the following information, where applicable:

- Accession codes, unique identifiers, or web links for publicly available datasets
- A description of any restrictions on data availability
- For clinical datasets or third party data, please ensure that the statement adheres to our policy

For qPCR experiments, reference genes were GAPDH (Fig. 1a-b) and RPL13A (Fig. 6d). For ddPCR experiments, reference gene was RNase P. For western blot analysis, loading controls were beta actin (Fig. 1 c-d; 2 d-f; supplementary fig. 1 a-d) and Na+/K+ ATPase (Fig 6 e-j).

The raw data generated in this study are provided in the Source Data file.

Human research participants

Policy information about studies involving human research participants and Sex and Gender in Research.

Reporting on sex and gender	Age and gender of patients and healthy donors from whom hNEC and hBEC were isolated are reported in the manuscript, according to data provided by the supplier (Epithelix, Plan-les-Ouates, Switzerland).
Population characteristics	Genotype of patients from whom hNEC and hBEC were isolated is reported in the manuscript, according to data provided by the supplier (Epithelix, Plan-les-Ouates, Switzerland).
Recruitment	N/A
Ethics oversight	Although this study has been approved by the Ethics Committee of the AOUI Verona (Approval No. 2917CESC), in this work all human samples were supplied by Epithelix (Geneva, Switzerland) as commercially available tissues (MucilAir). Thus, we did not utilized any other human sample.

Note that full information on the approval of the study protocol must also be provided in the manuscript.

Field-specific reporting

Please select the one below that is the best fit for your research. If you are not sure, read the appropriate sections before making your selection.

[x] Life sciences [ ] Behavioural & social sciences [ ] Ecological, evolutionary & environmental sciences

For a reference copy of the document with all sections, see nature.com/documents/nr-reporting-summary-flat.pdf

Life sciences study design

All studies must disclose on these points even when the disclosure is negative.

Sample size	No sample size calculation was performed. A sample size of n=3 is commonly used in biological studies using cell cultures. However, we employed two primary human nasal cells (hNEC) and seven bronchial cells (hBEC) isolated from patients with CF. In addition, we employed a pool of hNEC obtained from 14 subjects and eight primary hBEC samples. All human samples were commercially available from Epithelix (Plan-les-Ouates, Switzerland). ID of primary hNEC and hBEC with clinical and genetics characteristics have been included in supplementary informations.
Data exclusions	No data were excluded from the analysis
Replication	Experiments were performed in triplicate, indipendedent biological repeats, unless specified in figure legends
Randomization	Since we only used cell culture, randomization was not applicable
Blinding	Since we only used cell culture, blinding was not applicable

Reporting for specific materials, systems and methods

We require information from authors about some types of materials, experimental systems and methods used in many studies. Here, indicate whether each material, system or method listed is relevant to your study. If you are not sure if a list item applies to your research, read the appropriate section before selecting a response.

Materials & experimental systems

n/a	Involved in the study
☐	☒ Antibodies
☐	☒ Eukaryotic cell lines
☒	☐ Palaeontology and archaeology
☒	☐ Animals and other organisms
☐	☒ Clinical data
☒	☐ Dual use research of concern

Methods

n/a	Involved in the study
☒	☐ ChIP-seq
☒	☐ Flow cytometry
☒	☐ MRI-based neuroimaging

Antibodies

Antibodies used

Mouse anti-CFTR 596 isotype IgG2b (Cod. A4, University of North Carolina, Chapel Hill, NC, Lot. 596TJ21062820220526 – application WB, dil. 1:2500); Mouse anti-CFTR 570 isotype IgG1 (Cod. A2, University of North Carolina, Chapel Hill, NC, Lot. 570TJ20151016 – application IF, dil. 1:200); Mouse monoclonal anti-β-actin peroxidase antibody (Cod A3854, Sigma Aldrich, St. Louis, MO, Clone AC-15, purified from hybridoma cell culture; Lot. #0000112945 – application WB, dil. 1:10000). Peroxidase AffiniPure Goat Anti-Mouse IgG (H+L) (Cod. 115-035-062, Jackson ImmnunoResearch, Cambridge, UK– application WB, dil. 1:10000); Rabbit polyclonal anti- ACE2 isotype IgG (Cod ab15348, Abcam, Cambridge, UK, Lot. GR33516009-1 – application WB, dil. 1:1000, Application IF, dil. 1:200); Anti-rabbit IgG, HRP-linked Antibody (Cod. #7074 Cell Signaling Technology, Danvers, Massachusetts, MA, Lot. 27 – application WB, dil. 1:10000). Mouse monoclonal anti Na+/K+ ATPase (F2) isotype IgG1 (Cod. sc514614, Santa Cruz Biotechnology, Dallas, TX, – Application WB, dil. 1:500); Mouse anti-NHERF1 isotype IgG1 (cod. 611161, BD Biosciences Transduction Laboratories Franklin Lakes, New Jersey, NJ – application IF, dil. 1:200); Goat anti-mouse Alexa-488 (Cod. A32723, Thermo Fisher Scientific, Waltham, MA – application IF, dil. 1:1000); Goat anti-mouse Alexa-594 (Cod. A11012, Thermo Fisher Scientific, Waltham, MA – application IF, dil. 1:1000); Rabbit anti-mouse Alexa-594 (Cod. A27027, Thermo Fisher Scientific, Waltham, MA – application IF, dil. 1:1000); Mouse monoclonal anti-ACE2 E11 isotype IgG1 (Cod. sc-390851, Santa Cruz Biotechnology, Santa Cruz, CA – Application PLA, dil. 1:200); Rabbit anti-SARS-Cov-2 protein isotype IgG (cod. NBP3-11940, Novus Biological, Bio-Techne SRL, Milan, Italy –Application PLA, dil. 1:200). Rabbit monoclonal Recombinant anti-SARS-CoV-2 Spike RBD antibody, isotype IgG, Clone: HL1003 (Cod. ab281303, Abcam, Cambridge, UK, Lot. GR3412303-4 – Neutralization Spike, dil. 1:333);

Validation

All antibodies were commercially available and were validated by the manufacturer.
Anti-CFTR 596-A4 and 570-A2 (domain recognized: R domain was performed by the CFF (Bethesda, MD) and previously reported (Gentzsch et al., 2003. DOI: 10.1091/mbc.E04-03-0176). Rabbit polyclonal anti-ACE2 was validated in knock-out cell lines and tested in human testis, lung, intestine, brest and kidney lysates by the manufacturer (Abcam, Cambridge, UK). No cross reactivity to ACE1 (Ref. Zhu Y. et al., NatCommun 2021 DOI: 10.1038/s41467-021-21213-4). Mouse anti-rabbit IgG-HRP-coupled secondary antibody was tested in HeLa Hep G2, A549, JAr, NIH/3T3 and K562 (Ref. Ndeh D. et al. NatCommun 2020. DOI: 10.1038/s41467-020-14509-4). Mouse monoclonal anti-Na+/K+ ATPase alpha-1 (F2) specific for an epitope mapping between aminoacids 54-76 near the N-terminus of Na+/K+ ATPase alpha1 of human origin was validated by the manufacturer (Santa Cruz Biotechnology, Santa Cruz, CA) and tested on HeLa, Jurkat, SK-MEL-24 (Cross reactivity: mouse, rat, human, monkey)(Ref. McDonough A.A. et al. FASEB J. 1990. DOI: 10.1096/fasebj.4.6.2156741). Mouse monoclonal anti-NHERF1 was validated by the manufacturer (BD Transduction Laboratories, Franklin Lakes, NJ) and tested in human endothelial cells (No cross-reactivity) (Ref: Reczek D. et al. JBiolChem 1998. DOI: 10.1074/jbc.273.29.18452). Mouse monoclonal anti-ACE2 (E11 clone) was generated against ACE2 amino acids 631-805 of human origin and validated by the manufacturer in 293T cells transfected with or without human ACE2 and tested in human, mouse, and rat tissues (Santa Cruz Biotechnology, Santa Cruz, CA) (Ref. Zeng F. et al. EMBO Mol Med 2022 doi: 10.15252/emmm.202114844). Rabbit polyclonal anti-SARS-CoV-2 S protein was generated against amino acids 650-700 of SARS-CoV-2 Spike protein. Validation on SARS-CoV-2 Spike variants (alpha, beta, gamma, delta and omicron) was provided by the manufacturer. The antibody was tested in 293T cells transfected with Spike variants by immunofluorescence (cod. NBP3-11940, Novus Biologicals, Bio-Techne SRL, Milan, Italy, application PLA). Mouse monoclonal anti-β-actin peroxidase antibody clone AC15 was validated by the manufacturer (Sigma Aldrich, St. Louis, MO) and tested in human fibroblasts and chicken fibroblasts (Cross reactivity: sheep, carp, feline, chicken, rat, mouse, Hirudo medicinalis, rabbit, canine, pig, human, bovine, guinea pig (Ref: Eschenburg G. et al. Cancer Res 2012. doi: 10.1158/0008-5472.CAN-11-4072). Rabbit monoclonal Recombinant anti-SARS-CoV-2 Spike RBD antibody was validated with different applications, such as ICC, ELISA sandwich, Neutralizing and Indirect ELISA. Immunogenicity: Recombinant fragment within Human coronavirus SARS-CoV-2 Spike RBD (Strain Wuhan-Hu-1).

Eukaryotic cell lines

Policy information about cell lines and Sex and Gender in Research

Cell line source(s)

16HBE14o- and their clones W1282X- and G542X-CFTR were supplied by the Cystic Fibrosis Foundation (CFF, Bethesda, MD). CFBE41o- and their over-expressing models (F508del and WT) were provided by Dr. J.P. Clancy (CFF, Bethesda, MD). Calu-3 cells (ATCC, Manassas, VA) and their clones SH3 and Alter were provided by Dr. M. Chanson (University of Geneva, Switzerland) and were used as previously reported (Scheckenbach et al, 2011).
VeroE6 cell lines were purchased by ATCC (Manassas, VA). Validation and quality control were performed by the manufacturer.

Authentication

The commercially obtained cell lines were authenticated by suppliers. CFBE41o- and 16HBE14o- parental cell lines were generated by Gruenert D. (University of California, San Francisco). 16HBE14o- were edited at the endogenous CFTR locus using CRISPR/CAS9 to create isogenic cell lines W1282X. During immortalization of parental cells 16HBE14o-, SV40 sequence was inserted into one of the two CFTR alleles in these cells, rendering them mono-allelic with respect to functional CFTR expression. Full SV40 sequence information and whole genome sequencing data for CFBE41o- and 16HBE14o- parental cell

	lines are available upon request at the CFF (https://www.cff.org/cell-model-resources)(Ref: Cozens et al. Am J Respir Cell Mol Biol. 1994. DOI: 10.1165/ajrcmb.10.1.7507342; Valley HC. J Cyst Fibros. 2019. DOI: 10.1016/j.jcf.2018.001). Quality control on VeroE6 and Calu-3 cells were performed by ATCC (Manassas, VA). Alter and SH3 cells were not authenticated and were maintained under puromycin selection pressure as indicated in methods and previously reported (Scheckenbach et al, 2011). Weekly check of CFTR expression in SH3 and Alter was performed internally and reported in the manuscript.
Mycoplasma contamination	All cell lines were tested negative for Mycoplasma
Commonly misidentified lines (See ICLAC register)	No commonly misidentified cell lines were used in this study

Clinical data

Policy information about clinical studies

All manuscripts should comply with the ICMJEguidelines for publication of clinical research and a completedCONSORT checklist must be included with all submissions.

Clinical trial registration	N/A
Study protocol	Approval 2917CESC (Ethics Committee of the Azienda Ospedaliera Universitaria Integrata, Verona, Italy)
Data collection	Clinical data (gender, age, and genetics) of human samples (hNEC and hBEC) were supplied by Epithelix (Plan-les-Ouates, Switzerland) and have been indicated in the supplementary information.
Outcomes	Reduced levels of ACE2 in CF cells
